# Trehalose-6-phosphate and SnRK1 kinases in plant development and signaling: the emerging picture

**DOI:** 10.3389/fpls.2014.00119

**Published:** 2014-04-01

**Authors:** Allen Y.-L. Tsai, Sonia Gazzarrini

**Affiliations:** Department of Biological Sciences, University of TorontoToronto, ON, Canada

**Keywords:** sugar, trehalose-6-phosphate, SnRK1, plant development, seed maturation and germination, flowering, hormones, ABA

## Abstract

Carbohydrates, or sugars, regulate various aspects of plant growth through modulation of cell division and expansion. Besides playing essential roles as sources of energy for growth and as structural components of cells, carbohydrates also regulate the timing of expression of developmental programs. The disaccharide trehalose is used as an energy source, as a storage and transport molecule for glucose, and as a stress-responsive compound important for cellular protection during stress in all kingdoms. Trehalose, however, is found in very low amounts in most plants, pointing to a signaling over metabolic role for this non-reducing disaccharide. In the last decade, trehalose-6-phosphate (T6P), an intermediate in trehalose metabolism, has been shown to regulate embryonic and vegetative development, flowering time, meristem determinacy, and cell fate specification in plants. T6P acts as a global regulator of metabolism and transcription promoting plant growth and triggering developmental phase transitions in response to sugar availability. Among the T6P targets are members of the Sucrose-non-fermenting1-related kinase1 (SnRK1) family, which are sensors of energy availability and inhibit plant growth and development during metabolic stress to maintain energy homeostasis. In this review, we will discuss the opposite roles of the sugar metabolite T6P and the SnRK1 kinases in the regulation of developmental phase transitions in response to carbohydrate levels. We will focus on how these two global regulators of metabolic processes integrate environmental cues and interact with hormonal signaling pathways to modulate plant development.

## INTRODUCTION

As sessile organisms, plants rely heavily on developmental regulation as a mechanism to respond to environmental changes. As a consequence, plant development is much more plastic compared to animal development. Whereas an animal usually develops a predefined number of organs at predetermined time points, a plant can alter its rate of growth and the number and size of organs in response to endogenous and environmental stimuli at almost any point of its life cycle. In addition to their essential role as energy sources and structural components of cells, carbohydrates play equally important roles in signaling (Rolland et al., 2006; Eveland and Jackson, 2012; Lastdrager et al., 2014). Trehalose is a non-reducing glucose disaccharide, which accumulates to a high level in fungi, bacteria and non-vertebrate animals and plays a role as an osmoprotectant and carbon reserve (Elbein et al., 2003; Paul et al., 2008). With the exception of a few desiccation-tolerant resurrection plants, trehalose is found in very low amounts in plants; thus, a role as protectant or carbon storage seems less plausible (Avonce et al., 2006; Fernandez et al., 2010).

**FIGURE 1:**
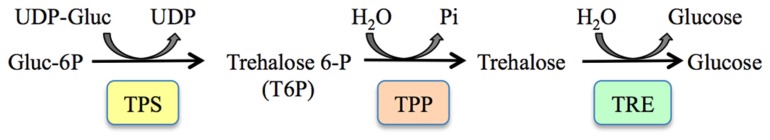
**Trehalose metabolism.** Trehalose-6-phosphate synthase (TPS) converts glucose-6-phosphate and UDP-glucose into trehalose-6-phosphate (T6P). T6P is dephosphorylated into trehalose by trehalose-6-phosphate phosphatase (TPP), and then hydrolyzed into two glucose molecules by trehalase (TRE).

In the last decade, several studies have highlighted the role of the precursor of trehalose, trehalose-6-phosphate (T6P), as an important signaling molecule (**Figure 1**). Most of our understanding on the role of T6P in plants came from the analyses of plants with altered T6P levels (Schluepmann and Paul, 2009; Ponnu et al., 2011; Schluepmann et al., 2012; O’Hara et al., 2013). T6P levels closely follow those of sugars, and an increase in sucrose results in a raise in T6P leading to metabolic changes to promote growth and development (Schluepmann et al., 2004; Lunn et al., 2006; Martínez-Barajas et al., 2011; Wingler et al., 2012). T6P is synthesized from glucose-6-phosphate and UDP-glucose by trehalose-6-phosphate synthase (TPS; **Figure 1**). T6P is then converted into trehalose by trehalose-6-phosphate phosphatase (TPP), and then hydrolyzed into two glucose molecules by trehalase (TRE). The *Arabidopsis* genome contains 11 *TPS,* 10 *TPP*, and 1 *TRE* (Leyman et al., 2001; Lunn, 2007). Yeast (*Saccharomyces cerevisiae*) complementation studies using mutants affected in TPS or TPP activities indicate that only TPS1 is an active TPS enzyme, while all *TPP* genes encode functional TPP proteins (Blázquez et al., 1998; Vogel et al., 1998; Ramon et al., 2009; Vandesteene et al., 2012). All *TPS* and *TPP* genes show a wide expression pattern throughout development, from embryos to leaves and flowers, although their exact functions are unknown (van Dijken et al., 2004; Paul et al., 2008; Ramon et al., 2009; Gómez et al., 2010; Vandesteene et al., 2012). Active TPSs and TPPs have also been isolated in monocots (Pramanik and Imai, 2005; Satoh-Nagasawa et al., 2006; Shima et al., 2007; Zang et al., 2011).

The mechanism by which T6P regulates growth and development is largely unknown, however, recent studies have shown that T6P inhibits the activity of the Sucrose-non-fermenting1-related kinase 1 (SnRK1) in monocots and dicots, suggesting it may be a conserved mechanism in plants (Zhang et al., 2009; Debast et al., 2011; Martínez-Barajas et al., 2011; Nunes et al., 2013). SnRK1 is a serine-threonine protein kinase homolog of the yeast Snf1 and animal AMPK. SnRK1/Snf1/AMPK kinases act as sensors of energy level in all eukaryotes and are activated under conditions of energy depletion or metabolic stress to inhibit growth and conserve energy for cell survival (Hardie, 2007; Baena-González and Sheen, 2008; Hedbacker and Carlson, 2008; Halford and Hey, 2009; Ghillebert et al., 2011; O’Hara et al., 2013). In plants, SnRK1 is activated by sugar depletion and under conditions of energy deficit including darkness and hypoxia (Baena-González et al., 2007). Once activated, SnRK1/Snf1/AMPK upregulate catabolism and downregulate anabolism to maintain energy homeostasis. Processes such as storage compound mobilization and autophagy are promoted to recover an energy deficit, while energetically demanding processes such as protein translation and cell proliferation are inhibited. Thus, SnRK1 activation signals low energy and low carbon levels, conditions opposite to those signaled by T6P. Accordingly, transcriptomic studies show that T6P and SnRK1 act as global regulators of gene expression, coordinating energy availability with plant growth in an opposite manner (Baena-González et al., 2007; Zhang et al., 2009).

Besides playing a well-established role in metabolism, SnRK1/Snf1/AMPK also regulate various aspects of development, which is perhaps not surprising considering their role in energy deficit response (Hardie, 2007; Baena-González and Sheen, 2008; Hedbacker and Carlson, 2008; Hardie, 2011; O’Hara et al., 2013). Some of these regulatory mechanisms are conserved between yeast and animals and may be associated with their regulation of energy balance. However, plants have also evolved unique mechanisms to regulate SnRK1 function by recruiting signaling molecules such as T6P and plant hormones, abscisic acid (ABA) in particular. The roles of the trehalose pathway and SnRK1 complex in metabolism and stress responses have been covered by several reviews (Baena-González and Sheen, 2008; Halford and Hey, 2009; Schluepmann and Paul, 2009; Fernandez et al., 2010; Ponnu et al., 2011; Schluepmann et al., 2012; O’Hara et al., 2013). This review will discuss the contrasting roles of T6P and SnRK1 in the regulation of developmental phase transitions and how these global metabolic regulators integrate endogenous and environmental signals to modulate plant development.

## T6P AND SnRK1 IN SEED DEVELOPMENT, GERMINATION AND ABA SIGNALING

### T6P REGULATES SEED MATURATION, GERMINATION, AND ABA SENSITIVITY

Development of complex organisms often occurs in multiple stages and is accompanied by dramatic morphological and physiological changes. Thus, the timing of expression of different developmental programs is carefully orchestrated and regulated in response to internal and external signals. In plants, two major developmental phase transitions occur; the transition from embryonic to vegetative development, which coincides with germination, and the transition from vegetative to reproductive development, or flowering. Developmental phase transitions involve major developmental reprogramming and are energy consuming processes, thus require remobilization and appropriate allocation of nutrients including sugars. During mid-embryogenesis, after pattern formation is completed, the embryo enters a phase of maturation during which cell division arrests and is followed by cell expansion, accumulation of storage reserves, acquisition of dormancy, and desiccation tolerance. Seed maturation is promoted by ABA and orchestrated by a network of transcription factors, including the B3-domain family proteins (Santos-Mendoza et al., 2008; Suzuki and McCarty, 2008; Nambara et al., 2010; Finkelstein, 2013). ABA levels peak during mid-embryogenesis to induce seed maturation processes, such as seed storage compounds accumulation, and then again during late embryogenesis to induce dormancy. The transition from cell patterning to maturation, which starts at the torpedo stage in *Arabidopsis*, is also accompanied by a decrease in glucose and a transient increase in sucrose levels, as well as an increase in *TPS1* expression (Gutierrez et al., 2007; Finkelstein, 2013). The *tps1* mutant showed delayed embryo growth, was arrested at the torpedo stage and had a higher sucrose level. The higher sucrose level and cell expansion defect of *tps1* could be partially rescued *in vitro* by reducing sucrose level (Eastmond et al., 2002). Physiological, anatomical and gene expression studies later showed that *TPS1* is required for the full accumulation of seed storage compounds, regulation of sugar levels and repression of starch synthesis during this transition (Gómez et al., 2006). The *tps1* defects could be rescued by expression of the *Escherichia coli* TPS gene (*otsA*), but not by trehalose supply, confirming that T6P is essential for embryo development and that it is the lack of T6P and not trehalose that leads to the embryonic phenotype (Eastmond et al., 2002; Schluepmann et al., 2003). These findings indicate that the increase in sugar level at the torpedo stage signals the transition from pattern formation to maturation through T6P, which regulates cell expansion and the accumulation of storage compounds during maturation (Eastmond et al., 2002; Gómez et al., 2006).

Further analysis of *tps1* mutants indicated *TPS1* is also required to promote germination and vegetative growth, and negatively regulates ABA signaling. Although the *tps1* embryo failed to germinate even after prolonged culture on reduced sugar, 30–40% of the seeds could eventually germinate after stratification, suggesting they may have increased dormancy. After germination, seedlings grew very slow and entered senescence even before flowering (Gómez et al., 2006). Weak alleles of *tps1,* or* tps1* rescued during embryogenesis using a seed-specific promoter (*ABI3:TPS1*) or by transient expression of *TPS1* (*GVG:TPS1*) also showed delayed germination and slow vegetative growth, supporting a positive role for *TPS1* in the regulation of postembryonic development (van Dijken et al., 2004; Gómez et al., 2010). These phenotypes were accompanied by increased levels of sugars, sucrose in particular, as well as activation of ABA signaling genes resulting in sugar and ABA hypersensitive phenotypes (Gómez et al., 2010). Thus, plants with lower T6P levels due to impaired T6P synthesis have a higher sucrose level during embryogenesis and vegetative growth. This leads to an activation of ABA signaling, resulting in increased dormancy and delayed germination and vegetative development.

Cross-talk between carbohydrates and ABA signaling has been uncovered in many screens aimed to identify sugar signaling components during germination on high sugar concentrations. Exogenous supply of high sugars inhibits germination and seedling development by increasing ABA level, and the resulting sugar-ABA interaction at this stage of development may be due to the activation of late-embryogenesis programs and/or be part of a stress response (Gazzarrini and McCourt, 2001; Finkelstein and Gibson, 2002; Gibson, 2005; Ramon et al., 2008). Heterologous expression of *E. coli* *TPS1* and* TPP* genes in *Arabidopsis* further support the cross-talk between sugars and ABA signaling pathways. Seedlings with increased T6P level, due to ectopic expression of *Arabidopsis* (*35S:TPS1*) or *E. coli* (*35S:otsA*) *TPS*, displayed reduced sensitivity to glucose and ABA similar to mutants affected in ABA synthesis and signaling (Schluepmann et al., 2003; Avonce et al., 2004; Ramon et al., 2008). Insensitivity to glucose-induced growth arrest displayed by *35S:otsA/TPS1* seedlings is likely due to a lack of ABA accumulation, as WT germinated on high glucose showed ABA accumulation while *35S:TPS1* did not (Avonce et al., 2004). In contrast, seedlings with reduced T6P level, due to ectopic expression of *E. coli TPP* (*35S:otsB*), showed glucose hypersensitive phenotypes (Schluepmann et al., 2003). Accordingly, *Arabidopsis* seedlings overexpressing one of the *TPP* genes(TPPG) are hypersensitive to ABA, while the *tppg* mutant shows the opposite phenotype (Vandesteene et al., 2012). These studies indicate T6P promotes germination in response to sugar levels possibly by decreasing seed sensitivity to ABA. The inhibition of seed germination caused by excessive sugar supply is similar to the inhibition of seed maturation caused by higher sucrose level in the *tps1* mutant, both of which are due to activation of the ABA signaling pathway and an imbalance in sugars/T6P. Although an increase in sucrose leads to an increase in T6P and promotes developmental phase transitions, such as the transition to seed maturation and the transition to vegetative development (germination), an excessive increase in sugar, such as in the *tps1* mutant at the end of pattern formation or during germination on exogenous high sugars, inhibits phase transitions through ABA signaling. This inhibition may be partly dependent on SnRK1 activation (see below).

### SnRK1 PLAYS A POSITIVE REGULATORY ROLE IN SEED MATURATION AND ABA SIGNALING

**FIGURE 2:**
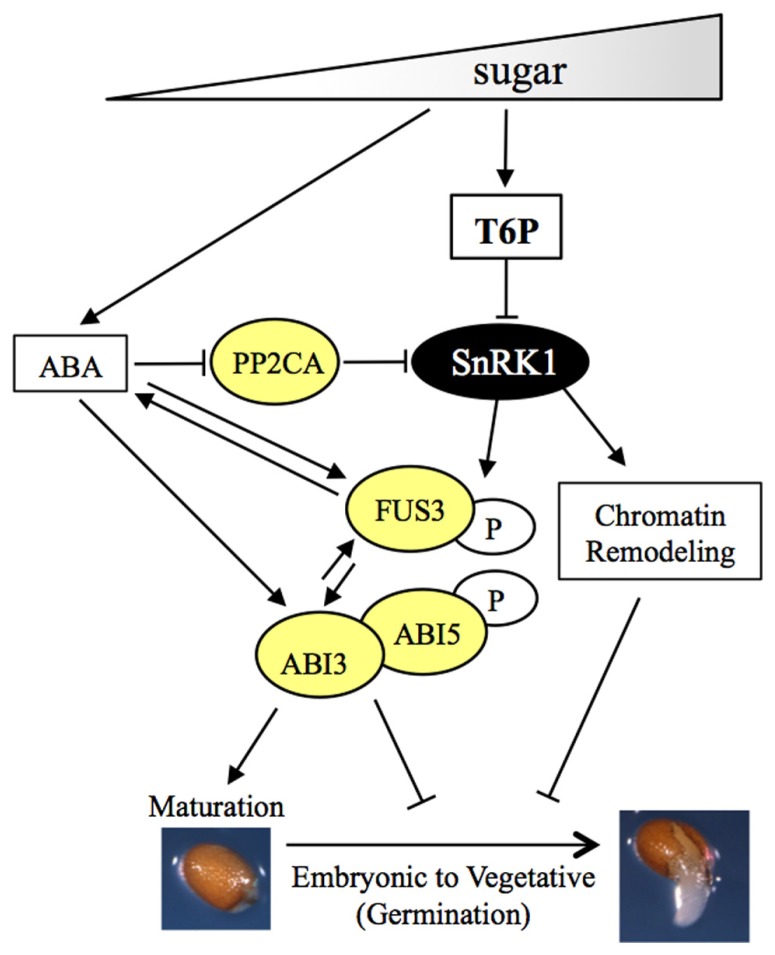
**Regulation of seed maturation and germination by sugars through T6P and SnRK1.** Model showing regulation of seed maturation and germination by sugars through T6P and SnRK1. Sugars promote the transition from pattern formation to maturation through activation of T6P and ABA synthesis and signaling pathways. An increase in ABA level during mid-embryogenesis, partly through the B3 domain FUS3, positively regulates SnRK1 by PP2C inactivation. T6P inhibits SnRK1 *in vitro* possibly to reset SnRK1 activity after ABA levels decline. SnRK1 modulates the activity or expression of B3-domain (FUS3, ABI3) and bZIP (ABI5) transcription factors through direct phosphorylation or transcriptional regulation to induce seed maturation and inhibit germination. ABA in turn positively regulates FUS3, ABI3, and ABI5 through transcriptional and/or post-translational regulation. SnRK1 regulates seed germination also through chromatin remodeling (see text for details).

Several lines of evidence indicate that SnRK1 regulates seed maturation processes and inhibits germination through positive regulation of ABA signaling. In pea, a 50–70% reduction of SnRK1 kinase activity using a *PsSnRK1-*antisense construct resulted in sucrose accumulation and maturation defects, including reduced conversion of sugars into seed storage compounds. Furthermore, a portion of seeds remaind green and germinated prematurely (Radchuk et al., 2006, 2010b). These phenotypes are reminiscent of mutants affected in B3-domain transcription factors, such as *abscisic acid insensitive3* (*abi3*) and *fusca3* (*fus3*), or mutants with reduced ABA synthesis or signaling (Finkelstein, 2013). In agreement with this, *PsSnRK1*-antisense plants have decreased ABA level and repression of *ABI3* (Radchuk et al., 2006, 2010b). Furthermore, in *Arabidopsis* SnRK1 phosphorylates and positively regulates FUS3; the latter promotes ABA synthesis and is itself positively regulated by ABA (Nambara et al., 2000; Gazzarrini et al., 2004; Tsai and Gazzarrini, 2012a). ABA positively regulates SnRK1 at the transcriptional level and also post-translationally, by recruiting and inactivating clade 2 Ser/Thr protein phosphatases (PP2Cs; Radchuk et al., 2010a,b; Rodrigues et al., 2013). Recently, SnRK1 has been shown to be dephosporylated and inactivated by PP2Cs, consistent with earlier studies showing SnRK1 dephosphorylation and inactivation by mammalian PP2Cs (Sugden et al., 1999; Rodrigues et al., 2013). PP2Cs are known to negatively regulate ABA signaling by dephosphorylating SnRK2 kinases, positive regulators of the ABA pathway, causing their inactivation (Cutler et al., 2010; **Figure 2**). Altogether, these findings indicate *SnRK1* is necessary for ABA-mediated seed maturation (**Figure 2**). Although SnRK1 activity can be inhibited in seeds *in vitro*, the mechanisms of SnRK1/T6P interaction in the regulation of seed maturation *in vivo* are currently unknown. Possibly, an increase in sucrose at the end of pattern formation induces ABA synthesis to promote maturation processes partly through SnRK1-mediated regulation of the B3 network, as well as by SnRK2-dependent pathways. An increase in sucrose also induces T6P accumulation, which could serve to inactivate SnRK1 after the ABA levels decrease. This would control the magnitude and duration of SnRK1 activity during maturation events. A similar mechanism has been proposed to regulate SnRK1 activity during recovery post stress (Rodrigues et al., 2013). It is also possible that SnRK1 expression and T6P accumulation may occur in different tissues and thus regulate these processes through different pathways spatially separated. Indeed, ABA modulation of seed maturation via SNF1 kinase appears to be restricted to the endosperm in Barley (Sreenivasulu et al., 2006). A spatio-temporal characterization of SnRK1 and T6P metabolic gene expression patterns is needed to better understand the T6P/SnRK1 interaction during seed maturation.

*SnRK1* overexpression delays seed germination, and genetic analysis showed that *FUS3* acts downstream of *SnRK1* (Tsai and Gazzarrini, 2012a). Besides increasing ABA synthesis, FUS3 also negatively regulate GA biosynthesis to prevent precocious germination (Nambara et al., 2000; Curaba et al., 2004; Gazzarrini et al., 2004). Thus, SnRK1 overexpression may affect seed maturation and germination by altering the ABA/GA ratio through FUS3. Interestingly, ABA and GA regulate the SnRK1 complex in an antagonistic manner; ABA induces while GA inhibits the expression of genes encoding different SnRK1 subunits (Bradford et al., 2003; Radchuk et al., 2010a,b). This indicates a feedback mechanism involving ABA regulation of the SnRK1 complex at multiple levels. In rice and wheat, however, mutations that reduce SnRK1 levels caused delayed germination. This has been linked to the indirect role of SnRK1 in the activation of α-amylases, which hydrolyze starch to sugars to sustain germination and seedling growth (Laurie et al., 2003; Lu et al., 2007). Thus, the mechanism of SnRK1 regulation of germination may differ between monocots and dicots and requires further investigation.

During germination, *SnRK1* overexpression causes hypersensitivity to high sugar and this hypersensitivity is dependent on ABA biosynthesis, as it can be rescued by inhibiting ABA biosynthesis pharmacologically or genetically. Accordingly, *35S:SnRK1* plants are hypersensitive to ABA (Jossier et al., 2009; Tsai and Gazzarrini, 2012b). Germination on high sugar induces SnRK1 phosphorylation by recruitment and inactivation of PP2C phosphatases by the ABA receptors (Jossier et al., 2009; Rodrigues et al., 2013). SnRK1 repression by PP2Cs is abolished in a quadruple *pp2c* knockout mutant, resulting in increased sensitivity to high concentrations of exogenous sugar during seedling establishment, while* PP2C* overexpression shows insensitivity (Rodrigues et al., 2013). Altogether, this suggests that ABA and SnRK1 signaling converge to arrest growth on high sugar. Although SnRK1 is typically repressed by sugars, SnRK1 activation during germination on high sugar has little to do with a physiological sugar response and instead may be part of a general stress response and/or result from activation of late-embryogenesis programs involving SnRK1 interaction with the ABA signaling pathway, as discussed above. This suggests SnRK1 and ABA signaling pathways may interact also during other stresses.

**FIGURE 3:**
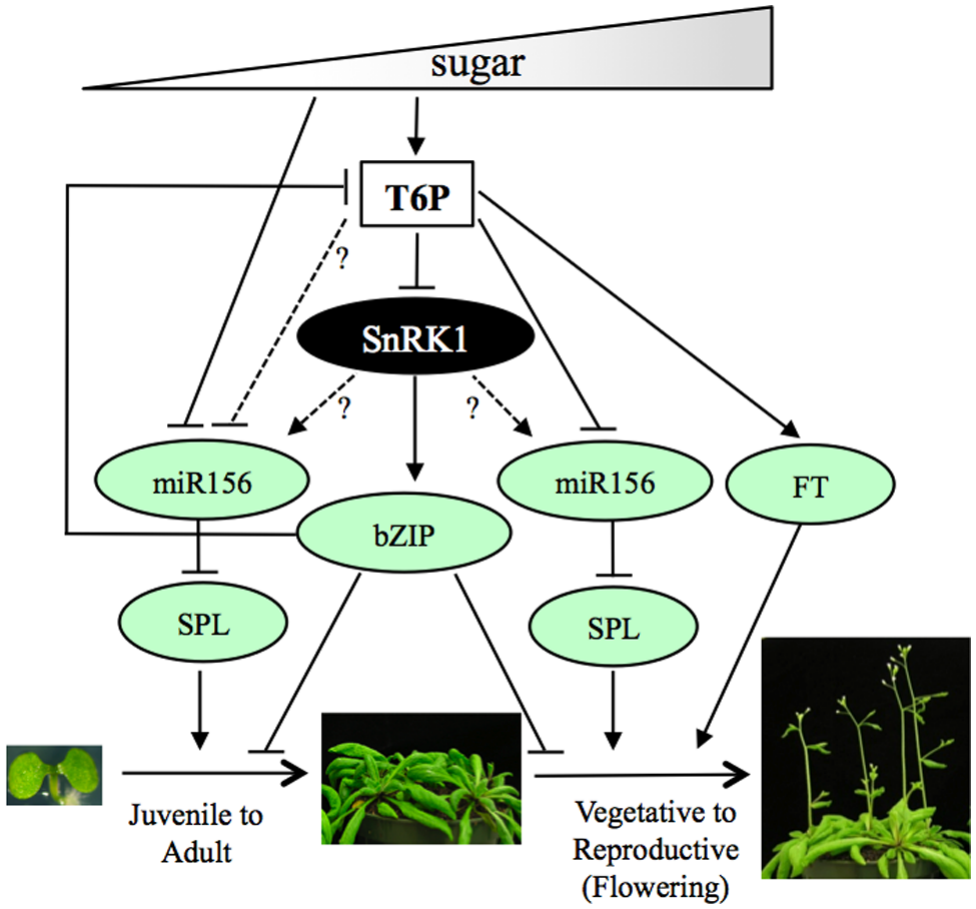
**Regulation of vegetative development and flowering by sugars through T6P and SnRK1.** Model showing regulation of vegetative development and flowering by sugars through T6P and SnRK1. Sugars regulate vegetative and reproductive developmental phase transitions through the antagonistic action of T6P and SnRK1. T6P inhibits SnRK1 activity in young leaves to promote growth, while SnRK1 delays vegetative growth and flowering in response to energy depletion through bZIP activation, which in turn represses T6P accumulation. T6P promotes flowering through the photoperiod pathway by activating *FT* in the leaves and *via* the miRNA156/SPL age pathways at the SAM by inhibiting miR156. Sugars also regulate vegetative phase change through the miR156/SPL pathway by repressing miR156. Question marks and dotted lines denote possible relationships between genes and/or signaling molecules (see text for details).

SnRK1s and SnRK2s share a similar kinase domain and phosphorylation motifs (Halford et al., 2003) and, not surprising, they have common substrates. Similar to SnRK2s, some SnRK1 substrates are transcription factors that mediate ABA signaling, such as the bZIP-type transcription factors ABA INSENSITIVE 5 (ABI5) and ENHANCED EM LEVEL (EEL/bZIP12), regulators of seed maturation and germination (**Figure 2**; Bensmihen et al., 2002; Lopez-Molina et al., 2002; Bitrián et al., 2011). Furthermore, both SnRK1s and SnRK2s phosphorylate ABA RESPONSIVE ELEMENT BINDING PROTEINS (AREBPs) or a target motif peptide derived from AREBP (Kobayashi et al., 2005; Furihata et al., 2006; Zhang et al., 2008; Fujita et al., 2013). This is consistent with expression studies showing S group bZIPs, including *bZIP11*, mediate SnRK1 signaling (Baena-González et al., 2007; Rodrigues et al., 2013). Interestingly, overexpression of the sugar repressible *bZIP11* inhibits plant growth by reprogramming metabolism, including reducing T6P level (Hanson et al., 2008; Ma et al., 2011). Accordingly, overexpression of *bZIP11* or SnRK1 rescues growth inhibition on high trehalose, which causes an increase in T6P level, suggesting cross regulation between SnRK1-bZIP and T6P (**Figure 3**; Schluepmann et al., 2004; Delatte et al., 2011). Thus, PP2Cs and bZIPs constitute points of convergence between ABA, SnRK1 and T6P signaling pathways and may integrate different signals (stress and energy level) to coordinate growth and development*.* Although T6P and SnRK1 play opposite roles during germination and in ABA signaling, the mechanisms of T6P-mediated inhibition of SnRK1 remain unknown (Zhang et al., 2009; Martínez-Barajas et al., 2011). Given the similarity in phenotypes displayed by seedlings with low T6P or high SnRK1 levels during germination on high sugar or ABA, and considering that T6P can inhibit SnRK1 activity *in vitro*, it will be interesting to test whether T6P inhibition of SnRK1 involves regulation of ABA signaling components.

## T6P AND SnRK1 IN VEGETATIVE DEVELOPMENT AND FLOWERING

### T6P REGULATES MERISTEM DETERMINACY

During vegetative and reproductive growth, lateral organ development relies on the activity of various meristems, which contain a population of highly organized and self-renewing stem cells. Axillary meristems produce secondary shoots or flowers and the determinacy of axillary meristem greatly affects inflorescence morphology and plant architecture overall (Eveland and Jackson, 2012*;* Tanaka et al., 2013). In maize, the trehalose pathway was shown to regulate inflorescence morphology by promoting determinacy of the axillary meristems. Loss-of-function mutations in *RAMOSA3* (*RA3*), which encodes a functional TPP enzyme, result in increased inflorescence branching (Satoh-Nagasawa et al., 2006). Two additional mutants were identified in maize, *ra1,* and *ra2*, showing a similar branching phenotype and found to affect C2H2 zinc finger and lateral-organ-boundary (LOB) transcription factors, respectively, with *RA2* and *RA3* acting upstream of *RA1* (Vollbrecht et al., 2005; Bortiri et al., 2006). *RA2* is expressed in the axillary meristems, while *RA1* and *RA3* are expressed in regions below the meristems and thus may regulate meristem determinacy by a non-cell autonomous signal (Vollbrecht et al., 2005; Bortiri et al., 2006; Satoh-Nagasawa et al., 2006; Tanaka et al., 2013).

Although the identity of the non-autonomous signal is unknown, sugars including T6P have been proposed to control meristem determinacy and inflorescence branching in the *RAMOSA* pathway (Satoh-Nagasawa et al., 2006). Loss of TPP activity in *ra3* would result in increased T6P level and convey a positive signal of energy availability to the meristem, thereby promoting development of axillary branches. In agreement with this, *Arabidopsis* plants expressing *E. coli* *TPS* (*35S:otsA*) have increased T6P levels and decreased apical dominance, while plants expressing the *E. coli* *TPP* (*35S:otsB*) have lower T6P levels and increased apical dominance (Schluepmann et al., 2003; van Dijken et al., 2004). However, single *tpp* T-DNA insertion mutants in *Arabidopsis* did not result in significant alterations of trehalose or T6P levels nor altered branching phenotype, possibly due to functional redundancy given the large family size and overlapping *TPP* expression profiles (Vandesteene et al., 2012). Analysis of higher order *tpp* mutants is necessary to shed light into the role of TPPs and T6P in axillary meristem function in *Arabidopsis*. In addition to a role for functional T6P enzymes in axillary meristem regulation, TPS members with undemonstrated enzymatic activity have also been shown to affect meristem determinacy. Although functionality of TPS6 in *Arabidopsis* has been controversial, the* tps6* loss-of-function and *TPS6* overexpression plants also showed altered apical dominance and branching in *Arabidopsis*. Thus, the mechanism of TPS6 regulation of meristem determinacy may possibly involve signaling properties for TPS6 (Chary et al., 2008; Ramon et al., 2009). Interestingly, the active rice TPS1 enzyme was shown to interact with the inactive TPS8 protein, suggesting the existence of TPS complexes and a possible regulatory role for select TPS proteins (Zang et al., 2011).

Insight into the mechanism of T6P regulation of meristem activity came from comparative analysis of WT and *ra* transcriptomes. Aside from a global alteration of energy production and sugar/trehalose metabolic pathways, altered TTP activity in *ra3* resulted in changes in the expression levels of transcription factors involved in developmental processes, as well as hormone sensing and signaling genes (Eveland et al., 2010). Recently, spatiotemporal expression profiles of maize *ra1* coupled with genome-wide occupancy of RA1 suggest RA1 may control meristem determinacy through modulation of GA synthesis and signaling, auxin biology, as well as by interacting with known regulators of stem cell maintenance, including KNOTTED1, and floral meristem identity genes such as LEAFY (Eveland et al., 2014). Interestingly, a *ra1* enhancer screen has identified *ramosa enhancer locus2* (*rel2*). *REL2* encodes a TOPLESS (TPL)-like co-repressor that interacts with RA1, suggesting meristem determinacy may be promoted by transcriptional repression mediated by the REL2/RA1 repressor complex (Gallavotti et al., 2010). In *Arabidopsis*, TPL interacts with the homeodomain transcription factor *WUSCHEL* (*WUS*), which is required for the maintenance of the shoot apical meristem, and negatively regulates auxin signaling (Kieffer et al., 2006; Szemenyei et al., 2008). Thus, the trehalose pathway may regulate meristem determinacy by integrating hormonal and sugar signals.

A possible mechanism of regulation of meristem activity by T6P is through modulation of cell division in response to sugar levels. Sugars have been shown to indirectly regulate cell proliferation by modulating the activity of important developmental regulators of meristem function. This was evident by the fact that exogenous sugar rescued the arrested meristem displayed by the *stimpy* mutant, which carries a mutation in *WUS*-*RELATED HOMEOBOX9 (WOX9),* a positive regulator of *WUS* (Wu et al., 2005). *STIMPY*/*WOX9* and sugar signaling induce cell proliferation and promote G2 to M transition in meristematic tissues by activating key cell cycle genes indirectly, through partial repression of the negative regulator *TPR-DOMAIN SUPPRESSOR* (Skylar et al., 2011). Recently, photosynthesis-derived glucose was shown to promote cell proliferation in the root meristem through activation of the transcription factor E2Fa by target of rapamycin (TOR) Ser/Thr protein kinase (Xiong et al., 2013). The TOR pathway is activated by nutrient abundance and triggers energetically expensive events, such as translation and cell proliferation to promote cell growth, thus acting in a similar way as T6P and antagonistically to the SnRK1 pathway (Robaglia et al., 2012; Dobrenel et al., 2013; Xiong and Sheen, 2014). Under nutrient limiting conditions, mammalian AMPK directly interacts with the TOR pathway by phosphorylating and inhibiting its upstream regulator TSC2 tumor suppressor and the TOR complex subunit regulator accessory protein of TOR (RAPTOR), while yeast Snf1 phosphorylation is negatively regulated by TOR (Inoki et al., 2003; Orlova et al., 2006; Hardie, 2007; Gwinn et al., 2008; Hardie, 2011). Thus, SnRK1 may also interact in a similar way with conserved components of the TOR pathway, such as RAPTOR. Finally, *Arabidopsis* SnRK1 has been shown to phosphorylate cell division inhibitors kip-related proteins (KRPs). However, phosphorylation of KRPs results in their inactivation (Guérinier et al., 2013). This contradicts AMPK established role as negative regulator cell division, as phosphorylation of p27^KIP1^ by AMPK promotes its stabilization inducing cell-cycle arrest (Williams and Brenman, 2008). Therefore, it will be important to determine whether and how regulation of cell proliferation and meristem activity by SnRK1 involves interaction with the TOR and/or other pathways.

### T6P, A POSITIVE REGULATOR OF VEGETATIVE GROWTH AND FLOWERING

Flowering time is controlled by complex interacting pathways that integrate environmental (light, temperature, day-length, or photoperiod) as well as endogenous (hormones, age) cues to ensure flowering occurs at the correct time for reproductive success (Amasino, 2010; Fornara et al., 2010). Flowering is an energy-consuming process that must be properly timed according to the nutritional status of the plant. Not surprising, nutrients including sucrose have been shown to regulate flowering time (Corbesier et al., 1998). During floral transition, an increase in sucrose results in a subsequent increase in T6P (Wahl et al., 2013). In turn, changes in T6P levels affect flowering time; a reduction in T6P level delays flowering, while an increase in T6P level promotes flowering. This was first evident in plants with higher T6P levels due to the expression of the *E. coli* *TPS* gene (*35S:otsA*), which caused early flowering (Schluepmann et al., 2003). In contrast, a reduction of T6P levels by artificial miRNA (*35S:amiR-TPS1)* or by expression of the *E. coli* *TPP* gene (*35S:otsB*) in *Arabidopsis* caused late flowering without affecting sucrose levels (Schluepmann et al., 2003; Wahl et al., 2013). In agreement with this, embryonic rescue of *tps1* with embryo specific (*ABI3:TPS1*) or dexamethasone-inducible (*GVG:TPS1*) promoters resulted in plants that flowered late or completely failed to flower even under inductive photoperiod (long days; van Dijken et al., 2004; Gómez et al., 2010). Alteration of flowering time by manipulation of T6P level was not due to a change in trehalose level, as expression of the *E. coli* trehalase gene (*35S:treF*), which converts trehalose in glucose, neither altered T6P content nor affected plant development (Schluepmann et al., 2003). Altogether, this data supports a role for T6P in promoting flowering in response to increasing carbohydrate levels.

Insights on how sugars regulate flowering time through T6P came from a recent study showing that T6P promotes flowering in response to changes in day length by activating the *FLOWERING LOCUS T* (*FT*; Wahl et al., 2013). Diurnal changes of T6P levels in leaves largely follow those of sucrose and peak at the end of the day when *FT* is induced in response to changes in the photoperiod. The mobile FT is made in the leaves and moves to SAM, where it triggers flowering only under inductive photoperiod (Corbesier et al., 2007; Turck et al., 2008). Expression of *FT* at the end of the day is reduced in plants with lower T6P level (*35:amiR-TPS1*) and almost absent in *tps1* mutant, but could be induced in *tps1* rescued by *GVG:TPS1*, suggesting T6P controls flowering by activating *FT* expression (Wahl et al., 2013). *FT* and T6P act in the same signaling pathway and T6P is upstream of *FT*, as shown by the rescue of *35:amiR-TPS1* late flowering by overexpression of *FT.* Accordingly, a reduction of T6P level in *35:amiR-TPS1* does not alter the late flowering phenotype of the strong* ft-10* allele under long days. Thus, the T6P pathway integrates into the photoperiod pathway to ensure plants flower at the correct time of the year and under appropriate carbohydrate levels (**Figure 3**).

Trehalose-6-phosphate is also sufficient to induce flowering at the SAM independently of the photoperiod pathway. This was evident in the *tps1* *GVG:TPS1* mutant, which is late flowering even under non-inductive conditions, in contrast to *ft-10* (Wahl et al., 2013). *TPS1* is expressed in the SAM where sucrose and T6P levels increase during the transition to flowering. Changes in T6P levels in the SAM, by expressing T6P synthesis (*CLV3:TPS1*) or catabolic (*CLV3:otsB/TPP*) genes, can alter flowering time independently of the photoperiod. Furthermore, *CLV3:TPS1* rescues the late flowering phenotype of *ft-10* suggesting T6P acts through FT in the leaves, but independently of FT at the SAM. T6P regulates flowering at the SAM partly by inhibiting microRNA 156 (miR156) *via* the miR156-dependent age pathway (Wahl et al., 2013). miR156 regulates flowering in response to plant age and ensures plants flower even though inductive signals are absent (Wang et al., 2009). miR156 delays vegetative phase transition and flowering by negatively regulating the *SQUAMOSA PROMOTER BINDING PROTEIN-LIKE (SPL)* genes, which promote flowering at the SAM, and combined overexpression of miR156 and downregulation of *TPS1* (*35S:amiR-TPS1*) cause additive effects and failure to flower under inductive and non-inductive conditions (Wahl et al., 2013). All these findings suggest T6P integrates environmental (photoperiod) and endogenous/physiological (carbohydrate levels) signals in leaves and at the SAM to fine regulate flowering time through multiple pathways (**Figure 3**).

Endogenous and environmental cues also regulate the transition from juvenile to adult development, or vegetative phase change. Recent studies have linked sugar abundance with plant age. As plants grow, sucrose progressively accumulates in pre-existing leaves and is transported to the young leaf primordia, where it is hydrolyzed to glucose. Glucose inhibits miR156 transcription, allowing expression of *SPL* genes and transition to the adult phase of development (Wu et al., 2009; Yang et al., 2011, 2013; Yu et al., 2013). Thus, the miR156/SPL pathway regulates both vegetative and reproductive developmental phase transitions in response to sugar abundance. It is tempting to speculate that T6P may also regulate vegetative phase transitions through the miR156/SPL pathway (**Figure 3**).

### SnRK1 INHIBITS VEGETATIVE DEVELOPMENT AND FLOWERING

In *Arabidopsis*, SnRK1 negatively regulates vegetative growth, senescence and flowering in a manner opposite to T6P. Simultaneous silencing of both *SnRK1s* caused *Arabidopsis* to flower early, while overexpression of *SnRK1* delays vegetative growth, senescence, and flowering (Baena-González et al., 2007; Tsai and Gazzarrini, 2012a). Comparable phenotypes were also shown in moss (*Physcomitrella* *patens*), where a *ppsnf1a ppsnf1b* mutant lacking two SnRK1 homologues showed accelerated development and premature senescence (Thelander et al., 2004). In agreement with this, SnRK1s are expressed throughout vegetative development, including the meristem and young leaf primordia (Takano et al., 1998; Pien et al., 2001; Bradford et al., 2003; Fragoso et al., 2009). SnRK1 activity inversely correlates with age and is higher in younger leaves, which have lower sugar accumulation compared to older leaves, suggesting a decrease in SnRK1 activity is required to allow vegetative development and flowering. T6P can inhibit SnRK1 activity in young and growing tissues and may act as a signaling metabolite necessary to regulate growth in response to increasing sugar availability (Zhang et al., 2009). It will be interesting to test whether T6P regulation of SnRK1 during vegetative development also involves the miRNA156/SPL pathway, as suggested above (**Figure 3**). Recently, a subset of miRNAs repressed by darkness and misregulated in the *dcl1-9* mutant, which affects miRNA biogenesis, were shown to be repressed by *SnRK1* overexpression, suggesting SnRK1-mediated transcriptional reprogramming following energy depletion may include post-transcriptional regulation of target genes (Confraria et al., 2013). miR172 acts downstream miR156 to control epidermal cell identity during vegetative phase change (Wu et al., 2009). Recently, miR172 expression was shown to increase during conditions of low energy (darkness) in a SnRK1-independent manner (Confraria et al., 2013), suggesting SnRK1 does not regulate vegetative phase change through miR172. Although SnRK1 role in miR156 regulation has not been investigated yet, miR156 is a direct target of the SnRK1 substrate, FUS3, which is a negative regulator of early vegetative phase transition (Lumba et al., 2012; Wang and Perry, 2013). Thus, SnRK1 may indirectly modulate miR156 level through phosphorylation of FUS3 and other regulators of vegetative phase change.

Delayed growth and flowering of plants overexpressing *SnRK1* can be rescued by a *fus3-3* mutant (Tsai and Gazzarrini, 2012a). *FUS3-*overexpression delays vegetative growth and flowering by increasing ABA level, while repressing GA biosynthesis and ethylene signaling (Gazzarrini et al., 2004; Lumba et al., 2012). Therefore, SnRK1 may regulate post-embryonic development through regulation of hormone biosynthesis and signaling. ABA and GA also establish a feedback regulatory loop by controlling the level and/or activity of the SnRK1 complex components (Bradford et al., 2003; Radchuk et al., 2010a,b). Interestingly, ABI5 has been shown to inhibit floral transition by inducing the floral repressor *FLOWERING LOCUS C* (*FLC*), an integrator of the vernalization and autonomous pathways and repressor of *FT* (Wang et al., 2013). The vernalization pathway represses *FLC* to promote flowering in response to a cold period, while the autonomous pathway triggers flowering in response to endogenous signals and is independent of the environment (Amasino, 2010). *ABI5* acts upstream of *FLC* and can directly bind ABRE elements in FLC promoter (Wang et al., 2013). Since ABI5 can be phosphorylated by SnRK1 *in vitro*, ABI5 may integrate hormonal and energy signals to inhibit flowering. Although interaction between SnRK1 and ABA during flowering has not been directly investigated, ABA acts synergistically with SnRK1 to inhibit vegetative growth during energy stress (dark), suggesting ABA enhances SnRK1 signaling. Furthermore, PP2Cs are required to inhibit SnRK1 after metabolic stress conditions subside (Rodrigues et al., 2013). Possibly, this mechanism may be adopted to inhibit vegetative growth and flowering under various stress conditions and to allow a fast recovery post stress.

## CONCLUSION AND PERSPECTIVE

The findings reviewed here show that sugars regulate the timing of developmental phase transitions through T6P and SnRK1 by integrating environmental signals and interacting with hormonal pathways. Developmental phase changes involve extensive reprogramming of gene expression and are energy-consuming processes. T6P and SnRK1 play fundamental and contrasting roles in the regulation of developmental transitions in response to sugar levels. SnRK1 is activated under conditions of low sugar to inhibit growth and conserve energy, while T6P acts as reporter of energy status and promotes growth and development in response to increasing sugar levels. Although T6P action may work partly through inhibition of SnRK1, the mechanism of this inhibition is unknown. Interestingly, SnRK1 modulates the expression of a subset of *TPS* genes (Baena-González et al., 2007; Usadel et al., 2008), suggesting a possible feedback regulation between T6P and SnRK1. Considering the majority of TPS members (10 of 11) lack TPS enzymatic activities despite retaining the catalytic site, these dubious enzymes may actually have signaling roles, similar to non-enzymatic roles proposed for other fungal TPS enzymes, and plant HEXOKINASE (HXK) and SnRK1 (Ramon et al., 2008; Fernandez and Wilson, 2012; Danielson and Frommer, 2013). A similar dual role has also been proposed for TPP enzymes, such as RA3 in maize (Eveland et al., 2010). Further characterization of the role of all proteins in the T6P pathway is needed to tease apart metabolic from signaling functions.

Mounting evidence connects T6P and SnRK1 with ABA signaling, the latter representing a point of cross-talk between these two pathways. Inactivation of SnRK1 by PP2C phosphatases parallels findings in yeast and animals (Steinberg et al., 2006; Hedbacker and Carlson, 2008). In addition to PP2Cs, Snf1 is also inactivated by other Ser/Thr phosphatases such as PP1 and PP2A under conditions of high nutrient levels (Hedbacker and Carlson, 2008). Thus, it is possible that different phosphatases are also required to fully inactivate SnRK1/Snf1/AMPK kinases, and possibly integrate different signals to control their activity. The relationships between SnRK1, T6P, and other hormones are more elusive. Transcriptome analyses of plants with altered SnRK1 or T6P levels suggest T6P may interact with GA and auxin in the regulation of meristem function and possibly flowering, but this is still an open field.

Undoubtedly, SnRK1 kinases act as global regulators of metabolism and affect plant development at several levels through transcriptional and posttranscriptional regulation, however, so far only few substrates have been identified making it difficult to understand SnRK1 mechanism of action. A more systematic approach to screen for SnRK1 targets may be required to gain a thorough understanding of how SnRK1 affects plant growth and development. Current data also support a role for sugars and SnRK1 in the regulation of gene expression through chromatin remodeling. Histone acetyltransferase (HAC) activity, which is associated with increased gene expression, is required for sugar sensing during seedling establishment and also regulates the expression of the SnRK1 complex (Heisel et al., 2013). In rice protoplasts, SnRK1 directly binds chromatin during hypoxia possibly through chromatin-associated SnRK1 complexes (Cho et al., 2012). This parallels findings in animal and yeasts, where AMPK was shown to accumulate at target gene chromatin in the nucleus and activate transcription in response to stress by phosphorylating histone subunits, while Snf1p activates gene expression by recruiting HAC to modify histones (Lo et al., 2001; van Oevelen et al., 2006; Bungard et al., 2010; Abate et al., 2012). Snf1 also regulates acetyl-CoA homeostasis and global histone acetylation (Zhang et al., 2013). It will be important to determine whether this epigenetic regulation of gene expression by SnRK1 is conserved in plants and whether SnRK1-mediated chromatin remodeling may be a mechanism to regulate developmental phase transitions and adapt to stress conditions.

The role of SnRK1 in delaying plant growth and phase transitions under low energy relates to the central role of AMPK in caloric restriction, where reduction in energy consumption correlates to lifespan extension (Cantó and Auwerx, 2011). One likely candidate for this lifespan-regulating mechanism shared by AMPK and SnRK1 is TOR, a central regulator of nutrient and energy level, as reduced TOR signaling delays aging and prolongs lifespan (Cornu et al., 2013). Given the conservation of AMPK/SnRK1 and TOR pathways in plants, it will be important to determine how SnRK1 and TOR signaling pathways interact. Whether SnRK1 signaling acts by inhibiting the TOR pathway through phosphorylation of the conserved mTOR binding partner RAPTOR, as shown in animals, or by other mechanisms remains to be determined (Gwinn et al., 2008; Hardie, 2011). Using a chemical genetic screen, phosphatases, and kinases involved in mitosis and cytokinesis have been identified as AMPK substrates (Banko et al., 2011). Similar high-throughput screens could be used in plants to identify SnRK1 substrates and better understand SnRK1 role in cell division.

## Conflict of Interest Statement

The authors declare that the research was conducted in the absence of any commercial or financial relationships that could be construed as a potential conflict of interest.
